# Heterogeneous Collaborative Sensor Network for Electrical Management of an Automated House with PV Energy

**DOI:** 10.3390/s111211544

**Published:** 2011-12-09

**Authors:** Manuel Castillo-Cagigal, Eduardo Matallanas, Álvaro Gutiérrez, Félix Monasterio-Huelin, Estefaná Caamaño-Martín, Daniel Masa-Bote, Javier Jiménez-Leube

**Affiliations:** ETSI Telecomunicación, Universidad Politécnica de Madrid, Av. Complutense 30, 28040 Madrid, Spain; E-Mails: manuel.castillo@upm.es (M.C.-C.); e.matallanas@alumnos.upm.es (E.M.); felix.monasteriohuelin@upm.es (F.M.-H.); estefan@ies-def.upm.es (E.C.-M.); dmasa@ies-def.upm.es (D.M.-C.); jleube@etsit.upm.es(J.J.-L.)

**Keywords:** smart grid, smart metering, photovoltaics, demand-side management

## Abstract

In this paper we present a heterogeneous collaborative sensor network for electrical management in the residential sector. Improving demand-side management is very important in distributed energy generation applications. Sensing and control are the foundations of the “Smart Grid” which is the future of large-scale energy management. The system presented in this paper has been developed on a self-sufficient solar house called “MagicBox” equipped with grid connection, PV generation, lead-acid batteries, controllable appliances and smart metering. Therefore, there is a large number of energy variables to be monitored that allow us to precisely manage the energy performance of the house by means of collaborative sensors. The experimental results, performed on a real house, demonstrate the feasibility of the proposed collaborative system to reduce the consumption of electrical power and to increase energy efficiency.

## 1. Introduction

The permanent increase of the electric demand is a general constant around the world. The availability, cost and sustainability of the energy resources have caused instabilities in the supply and the production of energy in recent years. Moreover, environmental damages have shown the need for new energy models. Therefore, investments in new energy infrastructures and grid improvements must be achieved [1,2]. Governments are passing laws to improve this management by means of Demand-Side Management (DSM), which has been identified as one of the main strategies to be promoted in order to guarantee security of electrical energy supply in the European Union [3]. However, there is no common accepted definition for the term DSM. In this paper, DSM is defined as the actions that influence the way consumers use electricity in order to achieve savings and higher efficiency in energy use. The combination of DSM with an automatic control of the household demand leads to a new concept called Active Demand-Side Management (ADSM). Moreover, in this paper, ADSM is implemented together with new-generation PV hybrid technology (grid connected-type inverters with small-scale electricity storage and an automatic control of the grid interface) from which not only PV systems operators can profit, but also other consumers connected to the same grid (through cooperative strategies) and the grid itself (if the PV systems respond to signals coming from the operators of the distribution system).

ADSM can be integrated into a broader concept that takes into account all the elements of the Smart Grid, which in the future will consist of an interactive electric network, for generators and consumers [4]. The network should be bidirectional, in which the information and the energy must flow from generators to consumers and vice versa. However, the information processing as well as the system coordination and organization are not trivial task. It is needed to provide the system with certain level of intelligence which is allowed, not only through a remote control by the distributors/generators, but through a self-organization which enables a dynamic response in real time that contributes to flatten the aggregated curve on the grid.

For the proper implementation of intelligent networks, actions are necessary in different areas. On the one hand, a distributed network structure must be created, in which not only large generators are responsible for powering the system, but micro-generators (located at the consumption areas) provide distributed generation, supplying local consumption. Therefore, the distributed generation reduces losses in transmission and distribution. On the other hand, it is necessary to incorporate a communication structure that allows users to adopt strategies of demand management based on information supplied by the operators of the grid [5].

In this scenario, Smart Meters come into play. They allow to obtain different information: the status of the local energy variables measured (*i.e.*, power and energy consumed at home), the status of the grid (e.g., alarms produced by the transformation center) or the price per kWh supplied by grid operator, between others. Smart Meters can receive orders from the operator to enable matching demand with the existing or planned energy generation [6–8]. Moreover, previous studies concludes that by offering consumption information to the users, the amount of energy demand at home can be reduced [9]. It is expected that the penetration of Smart Meters would grow during the next years in Europe and the United States [10,11]. The system presented in this paper is equipped with several Smart Meters in order to measure the power flows in the house. These meters offer different possibilities of remote monitoring and communication, providing added value to demand management. Collaborative sensors within the network allows the system to provide an efficient response to the needs of both the user and the grid. Nonetheless, complex billing calculations and load and storage management can be implemented. Furthermore, they can provide information from the grid to the users, making them aware of the instantaneous electric situation in the grid.

**Figure 1.**
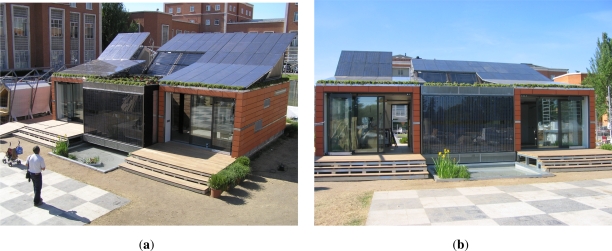
(**a**) Birds eye view and (**b**) south frontage of the “Magic Box” solar house.

The collaborative sensor network presented in this paper has been developed on a prototype of a self-sufficient solar house called “MagicBox” (see Figure 1). This house integrates sustainability elements based on the use of renewable energies, self-sufficiency energy methods and recycled construction materials. The electric self-sufficiency of “MagicBox” is based on consuming the maximum amount of energy at the same time it is produced, by means of a suitable control system. Moreover, the exploitation of its bioclimatic design reduces the energy needs to achieve adequate comfort levels inside the house [12]. It is an example of added value for PV electricity which arises from the combination of modern hybrid PV technology with a lead-acid battery storage system and DSM strategies in the residential sector. It consists of a 7 kWp PV generator, 7.5 kW grid connection, 9 kWh of lead-acid battery storage capacity with a 5 kWp battery inverter and electricity meters. It includes electrical appliances typical of a highly electrified home. The most consuming ones (*i.e.*, kitchen and laundry appliances) can be remotely controlled using Information and Communications Technologies (ICTs).

The remainder of this paper is as follows. Section 2 describes the architecture of the system. In Section 3, the communications protocols of the distributed sensor network are described. Section 4 presents the measurements carried out by the sensor network. In Section 5, a complete operation example is shown. Finally, Section 6 concludes the paper.

## 2. System Architecture

**Figure 2.**
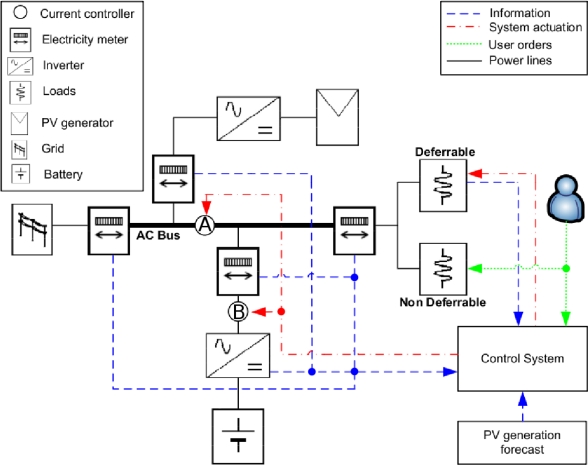
Depiction of the ADSM System architecture.

As aforementioned, the study presented in this paper has been carried out over a real house called “MagicBox”. It is equipped with a heterogeneous distributed sensor network involving measurements of different nature. The monitored variables depend on the different types of equipment that compose the system and the variables that define their operation. Moreover, there is an actuator network which allows to carry out any electrical energy management activity. A schema of the complete system presented in this Section is shown in Figure 2.

### 2.1. Heterogeneous Distributed Sensor Network

#### 2.1.1. Smart Metering

The house is equipped with four smart meters equipped with an RS-485 connection. Each smart meter is an energy monitoring device located at different points within the AC bus of the house. The system monitors the: (i) PV generation; (ii) grid exchange; (iii) house loads; and (iv) battery exchange. The meters offer power and energy measurements within a time interval which has been set to one minute. As aforementioned, the smart meters will be responsible, in the near future, to offer consumption profiles assigned to the house or specific requests from the network.

#### 2.1.2. Battery Inverter

The house is equipped with a storage system based on lead-acid batteries. It requires the presence of an inverter to convert AC to DC and vice versa. In addition, the inverter implements a variety of low-level battery management algorithms in order to increase its life-time and avoid electrical risks. It has also several sensors, which can be monitored by an external system, that obtain information about the status of the storage system.

#### 2.1.3. PV Generation Forecast

The system is equipped with a forecast PV generation framework [13] based on meteorological data from the Spanish State Meteorological Agency. The forecast program estimates the energy produced by the photovoltaic system in the short term. It generates a 24 h forecast of hourly values. At the beginning of each day, the PV generation profile for the current day is available. This information is uploaded to an exchange information area where it is collected by the control system. The control system will use this information in order to define the demand management actions for the whole day.

#### 2.1.4. Appliances Monitoring

“MagicBox” includes typical electrical appliances of a highly electrified house. The washing machine, dryer, dishwasher, refrigerator, oven and hood are integrated in a home automation system. Because of the automation, these appliances can be monitored and controlled by a remote system. The remaining appliances are monitored but not controlled.

Some of the appliances involve an instantaneous use because of their operation mode (e.g., lights, TVs, computers). This instantaneous use is indicated by the user in real time as in a common dwelling, and its consumption pattern represents the user’s habits. Because of this behavior, we have defined two types of electrical demand: (i) Deferrable, as the energy demand that can be displaced along the day; and (ii) Non-Deferrable, as the energy demand that is not controllable and represents the instantaneous consumption.

### 2.2. Actuator Network

#### 2.2.1. Current Controllers

The system is equipped with two current controllers which are integrated in the battery inverter. The position of these limiters can be observed in Figure 2. Limiter A separates the local consumption (*i.e.*, loads and battery) from the local (*i.e.*, PV generation) and external (*i.e.*, the grid) energy sources. This limiter allows a maximum current of 32 A. Limiter B controls the battery charge current, with a maximum current of 22 A. The use of both limiters allows the control system to modify the electrical energy behavior of the house by controlling the power flows.

#### 2.2.2. Controllable Loads

As aforementioned, part of the demand can be deferrable and therefore controllable. By using the communication with the home automation system, as explained in Section 3, this demand can be displaced during the day. Therefore, it allows the system to modify the consumption behavior of the house according to the electric grid needs or the available local generation.

### 2.3. Control System and Exchange Information Area

The control architecture is responsible for generating the appliances scheduling and charging/discharging the battery, according to the generation predictions offered by the prediction subsystem, user commands and grid status. It is divided in two controllers: (i) battery controller; and (ii) ADSM controller. Section 5 shows a complete working example of the different controllers. However, a wider description of the controllers is presented in [14].

The exchange information area is a database in which the control system allows different sensors and actuators to store and retrieve information. In addition, this area allows the analysis of the overall system performance. This exchange information area intends for scalability. Any subsystem developed in the future will obtain and deliver information from other subsystems through it.

### 2.4. The User Interface

The user interface is responsible for asking the user which tasks must be executed within the next 24 h: the appliances to start, the programs to use (different settings depending on the appliance) and the preferred time interval in which each task must be executed (e.g., the washing machine must be started within 13:00 and 17:00, the dishwasher must be finished at 18:00). After performing the energy balance and scheduling the tasks, the system shows the timetable and energy expectations to the user. If the user accepts the proposal, the system continues with the execution of the programmed tasks along the day. After all tasks have been executed, the system provides the user with information about the performance and consumption of the tasks, both the estimated and real values.

## 3. Communication Protocol

Because the heterogeneous system includes several types of sensors, different communication protocols are involved on it. This section focuses on the communication protocols used by each type of sensors.

### 3.1. Smart Meter

**Figure 3.**
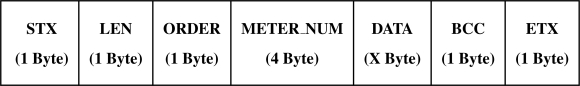
General structure of a transmission/reception frame.

The smart meters communicate through an RS-485 bus. The general structure of the frames is defined as a variable structure presented in Figure 3 and described as follows:

- **STX (0×02):** It is the Start Transmission byte. This character establishes the starting point of a data frame.
- **LEN:** It defines the length (number of bytes) of the frame.
- **ORDER:** It is the type of the data requested to the smart meter. In this paper, we only make use of two specific orders: (i) order number 34, to start the communication with the smart meter; and (ii) order number 6, to request the actual energy measurement.
- **METER_NUM:** It is the serial number of the meter.
- **DATA:** It is an array of bytes whose length and content is variable and dependent on the ORDER sent. The maximum length of this array is 200 bytes in both reception and transmission. For the starting communication frame (order 34) this field is the password of the meter, which is composed of four bytes. However, for the requested energy frame (order 6) this field returns a floating point value whose expression is represented as follows:
  (1) $${\left(-1\right)}^{S}\cdot 1.M\cdot 2^{\left(E-127\right)}$$
  where, *M* is the mantissa, *E* is the exponent and *S* is the signed bit.
- **BCC:** It is the Block Check Character and it is added to facilitate error detection. It consists of the arithmetic sum of the previous bytes.
- **ETX (0*×*03):** It is the End Transmission byte. This character establishes the end point of the data frame and indicates that the transmission is finished.

The communication with the meter starts with order number 34, in order to open the transmission channel between the meter and the computer. After opening the channel, we can request any data to the meter within the next five minutes, after which, the channel will be closed again. In the experiments performed in this paper, we only request the actual energy measurement to the meter (order 6) as explained in Section 4. All the meters are connected to the same RS-485 bus, so it is important not to send all the frames at the same time because of collisions in the communication bus.

### 3.2. Appliances

The communication with the deferrable loads has been carried through the home automation system. These devices incorporate Power Line Communication (PLC) modems that communicate with a Power Line Interface (PLINT) through the electrical network of the house [15]. The control system communicates with a gateway that directly accesses the PLINT. The communication is implemented through a webserver Application Programming Interface (API) required for the access to the PLINT. The different APIs allow us to get data from the appliances (e.g., actual status, program duration, time to end) and to send a series of basic commands (e.g., start the appliance, choose a program, pause or stop the program). It also has different callbacks that inform us about the events that occur during the operation of appliances (e.g., no water input, water tank full).

The communication with the server is through the standard Web Services Description Language (WSDL). WSDL is a language that provides a model for describing web services. The communication of the central computer with the home automation webserver is through Ethernet with a TCP/IP protocol. These APIs have been included in several communication libraries in order to simplify the appliances control.

### 3.3. Battery Inverter

The battery monitoring is done through the battery inverter. It includes all the necessary information from the battery for a proper operation mode of the battery controller through a TCP/IP communication with the inverter. The communication with the inverter is through the standard JavaScript Object Notation (JSON). JSON is a lightweight text-based open standard format designed for human-readable data interchange. The information requests and instructions are sent by a set of defined Remote Procedure Calls (RPCs).

## 4. Measurement

The different data obtained from the sensors will be interpreted by the control system. The measurement period has been set to one minute for all the sensors involved in the system. However, because of the complexity of each sensor, each source of information will cause a delay on the communication. Nonetheless, in order that the control system can analyze the energy flows in the house, the sensor data must be synchronous. This section explains how the different data are discretized and interpolated in order to allow a synchronous insertion in the exchange information area.

### 4.1. Smart Meters

After several implementations, we observed the smart meters have a random delay on the communication reply. Moreover, because all the meters are connected to the same bus, there is a difference in the time at which the measurement is obtained. This delay causes that the energy data received by each meter correspond to a different time and therefore, the control system cannot perform the energy balances in real time. Furthermore, different meters have different communication delays, which requires a measurement preprocessing to be created.

**Figure 4.**
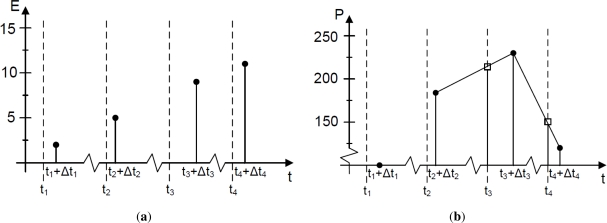
Meter data handling: (**a**) Energy measurement; and (**b**) power calculation.

Let us assume that the energy information is requested to a meter at a time step *t_i_*. Because of the communication delay, the system receives the information from the meter at the instant *t_i_* + Δ*t_i_*, where Δ*t_i_* represents the time delay (see Figure 4(a)). This delay needs to be taken into account in order to obtain a synchronization between all the meters. Therefore, the power at the response time must be calculated as:

(2) $$P\left[t_{i}+\Delta t_{i}\right]=\frac{\partial E\left[t\right]}{\partial t}=\frac{E\left[t_{i}+\Delta t_{i}\right]-E\left[t_{i-1}+\Delta t_{i-1}\right]}{T+\Delta t_{i}-\Delta t_{i-1}}$$

where *P*[*t_i_* + Δ*t_i_*] is the power at the response time, *E*[*t_i_* + Δ*t_i_*] is the energy data received from the meter, *E*[*t_i_*_–1_ + Δ*t_i_*_–1_] is the energy data received at the previous time step and *T* is the request period (*T* = *t_i_* *− t_i_*_–1_).

It is necessary to have the power measurements refer to the same time instant in order to perform the energy balances in real time. If we define *P*[*t_i_*] as the power consumption at the requested time, a first order interpolation can be implemented, as shown in Figure 4(b):

(3) $$P\left[t_{i}\right]=\frac{P\left[t_{i}+\Delta t_{i}\right]-P\left[t_{i-1}+\Delta t_{i-1}\right]}{T+\Delta t_{i}-\Delta t_{i-1}}\cdot \left(T-\Delta t_{i-1}\right)+P\left[t_{i-1}+\Delta t_{i-1}\right]$$

Moreover, we have observed that some communication frames are lost and therefore the energy information provided by the meter for that time interval is lost. If there is no energy information, we assume the same power value holds. Therefore, an inverse process is created, and we calculate the instant energy from the previous power value received.

(4) $$\begin{array}{l} \;\;\;\;\;\;\;\;\;\;\;\;\;P\left[t_{i}\right]=P\left[t_{i-1}\right]\\ P\left[t_{i}+\Delta t_{i}\right]=P\left[t_{i-1}+\Delta t_{i-1}\right]\\ E\left[t_{i}+\Delta t_{i}\right]=P\left[t_{i}+\Delta t_{i}\right]\cdot \left(T+\Delta t_{i}-\Delta t_{i-1}\right)+E\left[t_{i-1}+\Delta t_{i-1}\right] \end{array}$$

### 4.2. Appliances

The working status of each deferrable appliance can be received through the home automation system. This information contains the operating parameters such as the temperature, spin revolutions or type of program in execution. Moreover, the system provides time information such as the time required to run a specific program in the appliance or the remaining time to the end of the current task. However, there is no precise information for the non-deferrable loads; only general information of these loads is available through the smart meters.

As aforementioned, controllable or deferrable loads can be turned on or off remotely. Moreover, depending on the type of the load, it is necessary to supply some parameters to start the appliance. These parameters are indicated in Table 1.

### 4.3. Battery Inverter

The battery State of Charge (SoC) is the main parameter monitored from the battery inverter. It is a percentage value that indicates how much energy is available in the storage system with regard to its nominal capacity. The grid voltage is monitored too. The battery inverter is equipped with a very accurate AC voltage sensor. This information is stored every minute in the exchange information area so it is available for a future control system scheduling and to study the operation of the storage system during the experiments.

## 5. Application

The distributed sensor network described previously has been used to implement an energy management control system. The system has been divided in two main controllers: (i) battery controller and (ii) ADSM controller. The battery controller is responsible for managing the electricity energy flows in the storage system. The ADSM controller is responsible for the distribution of the energy demand throughout the day. Energy management can be focused on different criteria, in our case, the selected criterion is the self-consumption maximization. The term self-consumption on distributed generation electric networks focuses on the usage of the own generated energy, while the energy provided by the grid remains an optional generator or consumer [16]. In this section both controllers and the self-consumption evaluation are detailed. Moreover, a real experiment will show the potential of the aforementioned sensor network.

### 5.1. Self-Consumption Evaluation

In order to evaluate the electrical energy behavior of the system, hereafter we describe the system energy balances and define the self-consumption factor [16], which evaluates how much energy is supplied to the loads by local energy sources. The energy balances are the integral of the power flows during a time period (e.g., *E_PV,load_* is the energy which comes from the PV generation and is directly consumed by the loads). The time period of evaluation has been fixed to one day. The following equations are applied to the electrical energy balances.

For the total PV electrical energy:

(5) $$E_{\mathrm{PV}}=E_{\mathrm{PV},\mathrm{load}}+E_{\mathrm{PV},\mathrm{bat}}+E_{\mathrm{PV},\mathrm{grid}}$$

where *E_PV_* is the total PV electrical energy, *E_PV,load_* is the PV energy that fed the loads, *E_PV,bat_* is the energy stored in the battery and *E_PV,grid_* is the PV energy that is exported to the grid.

For the electrical demand:

(6) $$E_{\mathrm{Load}}=E_{\mathrm{PV},\mathrm{load}}+E_{\mathrm{Bat},\mathrm{load}}+E_{\mathrm{Grid},\mathrm{load}}$$

where *E_Load_* is the total energy consumed by the loads, *E_Bat,load_* is the energy imported form the battery to the loads and *E_Grid,load_* is the electrical energy taken from the grid to the load.

For the electrical energy managed in the battery:

(7) $$E_{\mathrm{Bat},E}=E_{\mathrm{Bat},I}+E_{\mathrm{SoC}}-E_{\mathrm{SoC},0}$$

where *E_SoC_* is the SoC at the end of the time period and *E_SoC,_*_0_ is the SoC at the beginning of the period.

Once the energy balances in the house have been defined, we will make use of the self-consumption factor previously defined in [16]. The self-consumption represents the electrical energy consumed by the loads which is supplied only by the local generation sources. Therefore, the self-consumption factor (*ξ*) is represented by Equation (8):

(8) $$\xi =\frac{E_{\mathrm{PV},\mathrm{load}}+E_{\mathrm{Bat},\mathrm{load}}}{E_{\mathrm{Load}}}$$

It should be noted that *ξ* can be used in different time-frames. Moreover, because *ξ* is normalized by the load demand (*ξ* ∈ [0, 1]), it allows to compare systems with different sizes and loads. *ξ* = 0 would be the case of a system with no local generation, and *ξ* = 1 when all the energy consumed is locally supplied.

### 5.2. ADSM Controller

The developed ADSM control system is part of the architecture previously shown in Figure 2. Its purpose is to take into consideration the operating logic of the system and to act on it when necessary. The ADSM control system actions are focused to meet two main objectives: (i) efficacy in service, in the sense of satisfying the demand of users; and (ii) optimize self-consumption. The sole possibility of meeting the aforementioned objectives is through the collaboration of users, under the assumption that they do not only want to satisfy their consumption needs, but will accept the rationalization of them as a function of the actual conditions in the global system. From this point of view, it is essential to design a communication system between the user and the control system. The user has to be informed about the status of the system so that he can participate in its demand rationalization. On the other hand, the control system should reduce the effort in the user’s decision-making, so it should have some autonomy conditioned to the user request and the actual energy status. Therefore, the user decisions are inputs of the control system.

As aforementioned, the ADSM control system can only act directly on the deferrable loads of the house. The user indicates which particular task should be scheduled and the time interval in which it should be executed. The control system uses the PV generation forecast and places the loads at the time of the day at which self-consumption is maximized. Non-deferrable loads cannot be moved because they involve an instantaneous use. However, the consumption information of the non-deferrable loads is provided to the user so that he can voluntarily modify their usage depending on his energy objectives.

### 5.3. Battery Controllers

In order to use the stored electricity, the house is equipped with a battery inverter. This inverter not only carries out the energy conversion but also allows to control the power flows in the house. By making use of these flow controllers, several high-level (software) battery controllers have been developed and tested. The objective of theses controllers is to maximize *ξ*. Their main characteristics are: (i) management of the battery inverters currents; (ii) avoidance of electricity exchange with the grid (they only charge the battery with PV generation excess and discharge the battery to supply the loads); and (iii) preservation of the battery against overcharge and overdischarge. The controller that performs best, in terms of maximizing *ξ*, will define the next states depending on the battery state of charge (SoC):

#### 5.3.1. Self-Consumption Operation

This battery controller state occurs when *SoC* ≥ 25 %. In this situation, grid-connection is physically maintained, but exchanges of electricity with the grid are minimized. The following equation explains its behavior:

(9) $$\begin{array}{l} \max\left(I_{A}\right)=I_{\mathrm{PV}}+I_{\mathrm{error}}\\ \max\left(I_{B}\right)=I_{\mathrm{PV}}-I_{\mathrm{Load}} \end{array}$$

where *I_A_* is the current through the current limiter A, *I_B_* is the current through the current limiter B, *I_PV_* is the current generated by the PV system, *I_Load_* is the current consumed by the loads and *I_error_* is the measurement error.

The current limiter A provided by the inverter has a measurement error, what can cause some malfunction in the ADSM controller. Moreover, this error is not constant in time, presenting a complex dynamics, so it has to be taken into account in real time in order to perform a correct control. Because the measurements carried out by the meters are correct, they can be used as reference. Therefore, the battery controller calculates the error and match the current limiter value with it. This effect has not been detected in the current limiter B.

#### 5.3.2. Overdischarge Operation

This battery controller state occurs when *SoC* ≤ 20 %. The battery stops supplying energy to the loads and the grid is asked to supply the energy left to the loads. At this situation, battery charging is only allowed if the PV generation exceeds the load demand:

(10) $$\begin{array}{l} \max\left(I_{A}\right)=\mathrm{MAX}\\ \max\left(I_{B}\right)=I_{\mathrm{PV}}-I_{\mathrm{Load}} \end{array}$$

where *MAX* is the maximum value of the current limiter A. It means that the AC line is totally open.

Moreover, there is hysteresis between the two states (20% ≤ *SoC* ≤ 25%), because it is necessary to avoid fast state changes.

**Figure 5.**
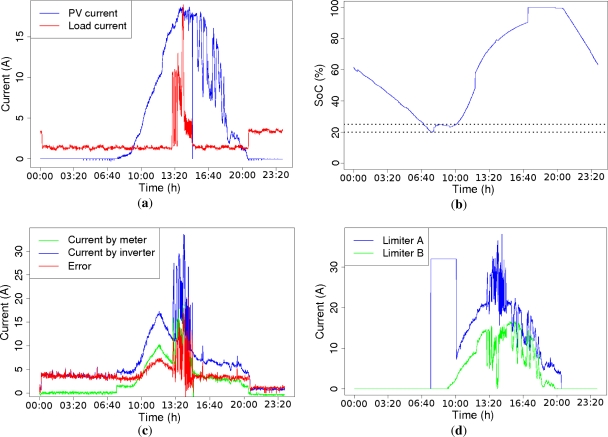
Battery controller example: (**a**) PV and Load current; (**b**) SoC; (**c**) current error of limiter A; and (**d**) current limits.

In Figure 5, a battery controller operation example is shown. *I_PV_* and *I_Loads_* are presented in Figure 5(a). They are the measurements obtained from the meters during an execution day. Clearly, the *SoC*(%) parameter changes the operation mode of the controller. Its evolution during the same day is depicted in Figure 5(b), the horizontal dotted lines mark the 20%–25% SoC level. Figure 5(c) shows the current measured by the meter and the battery inverter and the difference between them (error). Notice that the error fluctuations are considerable. By using this information the current limiters are set. Their behavior can be observed in Figure 5(d).

### 5.4. Operation Example

By making use of the distributed sensor network and the controllers previously explained, real experiments have been carried out in “MagicBox”. In this section we present an example in which we compare the importance of having some automated loads. The first experimental setup (ES1) is dedicated to a day of operation in which all the loads are considered non-deferrable, that is, the user does not allow the control system to adjust the loads at the more convenient time, from a self-consumption perspective. The second experimental setup (ES2) is dedicated to a day of operation in which some electrical appliances can be moved by the ADSM controller. Non-deferrable consumption profiles have been implemented based on studies of typical residential consumption [17–19]. In this example, the battery capacity has been fixed to 9 kWh and the deferrable consumption (in ES2) represents the 20% of the total demand [14].

**Figure 6.**
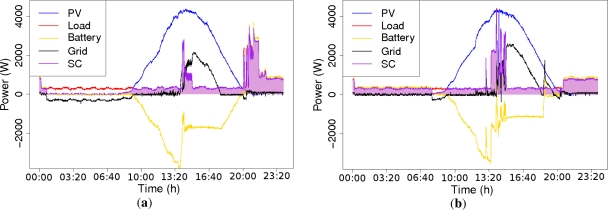
Complete operation example: (**a**) ES1 and (**b**) ES2.

Figure 6 shows the power flows for both experiments and the resultant self-consumed energy. The sign of the different power flows are as follows: the electricity demanded by the loads (in red) is positive, the generated electricity (in blue) is positive, the flow of power in the battery (in yellow) is positive when discharging to the loads and negative when charging from the excess of PV generation, and finally the power of the gird (in black) is negative when it supplies electricity to the loads and positive when the local energy excess is exported to the grid (in a full-charge situation and PV generation excess). The self-consumed electricity (in purple) represents the flow of demanded power that is supplied by local sources of energy. Notice that the electricity demanded (in red) agrees with the self-consumed electricity if there is enough PV electricity or energy in the battery, thus both lines are overlapped.

Figure 6(a) shows the power flows for ES1, where the loads are mostly concentrated in the evening (around 21:00). Before sunrise, the battery is able to supply the energy demanded by the loads. Once it is exhausted, the grid must supply the demanded energy. The batteries start being charged only when PV generation exceeds the local demand (remember that the batteries are only charged from PV generation excess). At lunch time (around 14:00) a fast increase of demand modifies the battery operation mode, which changes to discharge mode in order to supply the sharp peak demand without importing electricity from the grid. After the lunch time peak demand is finished, the PV electricity is again able to supply all the energy to the loads and the battery is charged again with the energy excess. In the evening, the system has to deal with the main consumption peak. At this time, the battery is discharged to supply the evening loads until all PV electricity stored is consumed. For this experimental setup, a value of *ξ* = 80% has been achieved.

Figure 6(b) shows results for ES2. We observe, the deferrable loads have been displaced, by the automatic ADSM system, to the maximum generation hours, in order to optimize self-consumption. In this experimental setup, batteries are used before sunrise to supply the existing load until the stored PV electricity of the previous day has been consumed. Later on, the batteries start being charged with the excess of PV power until lunch time (around 14:00), when the rapid increase of the demand occurs (not only cooking appliances are used, but also deferrable appliances) and the stored PV electricity is used to complement the existing PV generation in order to supply the demand without importing electricity from the grid. When the loads demand decreases below the PV generation, the excess is again stored in the batteries until there is no more generation. This experimental setup achieves a *ξ* = 97% value.

## 6. Conclusions

This paper has described a heterogeneous network of distributed sensors for monitoring and managing the energy performance in a solar house. This house is equipped with a PV system as local generation, a lead-acid battery storage system and a connection with the grid. Therefore, all the possible energy actions that a local system can perform take place: (i) generation; (ii) storage; and (iii) energy exchange.

We have implemented an exchange information area where the entire sensor network stores the measured data. Because of the communication delays, it is necessary to synchronize the data every minute. For the smart meters this synchronization takes on special significance as there is a random delay even within each sensor. The transformation of the energy value into power value requires knowledge of the communication delay in order to derive the energy. It is also necessary to interpolate the measurements of the smart meters to perform an energy balance of housing in real time.

An ADSM and a battery controller have been implemented, both using the sensor network information. The ADSM controller combines a new generation hybrid PV system with strategies of Demand Side Management performed by an intelligent control system. The system is able to displace the consumer load curve in response to local and external conditions, thus optimizing the PV use and enhancing the PV value for the user. The battery controller makes use of the current limiters maximizing self-consumption.

Smart meters are a reality in many countries around the world and they are nowadays experiencing a tremendous growth. Using this type of sensors, monitoring and control capabilities in both demand and supply of equipment for generation and local storage leads to a variety of energy strategies. These strategies will allow to meet the smart grids paradigm in a near future.

## Figures and Tables

**Table 1.** Appliances variables supplied by the user to the control system.

**Appliance**	**Variable 1**	**Variable 2**
Washing machine	Temperature	Spin revolutions
Dryer	Spin revolutions	Not used
Dishwasher	Washing parameter	Not used
Oven	Temperature	Cooking time
Hood	Fan speed	Light intensity
Refrigerator	Temperature	Not used
Freezer	Temperature	Not used
Air conditioning	Temperature	Cooling time
